# Supplementary material for: Linking peak intensity of mechanically stimulated bioluminescence and cell surface area in dinoflagellates
Source: Biol Open. 2025 Sep 19;14(9):bio062190. doi: 10.1242/bio.062190 (PMC12486207; doi:10.1242/bio.062190)
Supplement: Supplementary information [file biolopen-14-062190-s1.pdf]

Dataset 1.

Available for download at
https://journals.biologists.com/bio/article-lookup/doi/10.1242/bio.062190#supplementary-data

Dataset 2.

Available for download at
https://journals.biologists.com/bio/article-lookup/doi/10.1242/bio.062190#supplementary-data

Biology Open • Supplementary information
